# Evaluation of dosing strategy for pembrolizumab for oncology indications

**DOI:** 10.1186/s40425-017-0242-5

**Published:** 2017-05-16

**Authors:** Tomoko Freshwater, Anna Kondic, Malidi Ahamadi, Claire H. Li, Rik de Greef, Dinesh de Alwis, Julie A. Stone

**Affiliations:** 1 Department of Pharmacokinetics, Pharmacodynamics and Drug Metabolism, Merck & Co., Inc., 2000 Galloping Hill Road, Kenilworth, NJ USA; 2 Quantitative Solutions, a Certara Company, Kloosterstraat 9, Oss, The Netherlands

**Keywords:** Pembrolizumab, Fixed dose, Flat dose, Dosing strategy, Clinical dose, Exposure-response analysis, Population pharmacokinetics analysis

## Abstract

**Background:**

Traditionally, most monoclonal antibodies (mAbs) have been dosed based on body weight because of perceived contribution of body size in pharmacokinetic variability. The same approach was used in the initial pembrolizumab studies; however, following availability of PK data, the need for weight-based dosing for pembrolizumab was reassessed.

**Methods:**

A previously established population PK (popPK) model as well as exposure-response results from patients with advanced melanoma or non–small cell lung cancer (NSCLC) were used to evaluate the potential application of a fixed dosing regimen with the aim of maintaining pembrolizumab exposures within the range demonstrated to provide near maximal efficacy and acceptable safety. Individual PK exposures for the selected fixed dosing regimen from recently completed trials with head and neck cancer, NSCLC, microsatellite instability high (MSI-H) in colorectal cancer (CRC) and urothelial cancer were used to confirm acceptability. To determine whether fixed dosing would maintain exposures within the range of clinical experience, the individual AUC distributions with fixed dosing were compared with the range of exposures from the pembrolizumab doses that were evaluated in early studies (2 mg/kg Q3W, 10 mg/kg Q3W/Q2W).

**Results:**

Body-weight dependence of clearance was characterized by a power relationship with an exponent of 0.578, a value consistent with fixed- and weight-based dosing providing similar control of PK variability. A fixed dose of 200 mg Q3W was investigated in trials based on predicted exposures maintained within the established exposure range in all patients. Mean (% CV, n) AUC_ss, 6-weeks_ was 1.87 (37%, 830), 1.38 (38%, 760) and 7.63 (35%, 1405) mg*day/mL in patients receiving 200 mg, 2 mg/kg and 10 mg/kg Q3W pembrolizumab. High-weight patients had the lowest exposures with 200 mg Q3W; however, exposures in this group (>90 kg) were within the range of prior clinical experience at 2 mg/kg Q3W associated with near maximal efficacy.

**Conclusions:**

Doses of 200 mg and 2 mg/kg provide similar exposure distributions with no advantage to either dosing approach with respect to controlling PK variability. These findings suggest that weight-based and fixed-dose regimens are appropriate for pembrolizumab.

**Electronic supplementary material:**

The online version of this article (doi:10.1186/s40425-017-0242-5) contains supplementary material, which is available to authorized users.

## Background

Dosing of therapeutic monoclonal antibodies (mAbs) is often based on body size, operating from the perception that this reduces intersubject variability in drug exposure [1]. However, because of the specific properties of mAbs (selective mode of action, with substantial therapeutic window) and the advantages of fixed dosing (increased convenience, elimination of wastage, improved safety resulting from a reduced chance for dosing errors, and improved compliance), the weight-based dosing paradigm has recently been re-evaluated [1–3].

Pembrolizumab (Keytruda, Merck & Co., Inc., Kenilworth, NJ, USA) is a potent, humanized IgG4 monoclonal antibody against programmed death 1 (PD-1) receptor that directly blocks the interaction between PD-1 and its ligands, PD-L1 and PD-L2. Pembrolizumab has demonstrated robust, durable antitumor activity and a manageable safety profile against several advanced malignancies. Early clinical studies of pembrolizumab employed a body-weight–based dosing strategy of 2 mg/kg every 3 weeks (Q3W) to 10 mg/kg every 2 weeks (Q2W), but in more recent trials a fixed-dose regimen (fixed with respect to body weight) has been introduced. In this paper, the analyses used to evaluate weight-based vs fixed dosing for pembrolizumab are described and the basis for the decision to switch to fixed-dose regimens in clinical trials is discussed. Results from clinical investigations of 200 mg Q3W are provided as confirming the resulting exposures of the selected fixed dose.

**Fig. 1:**
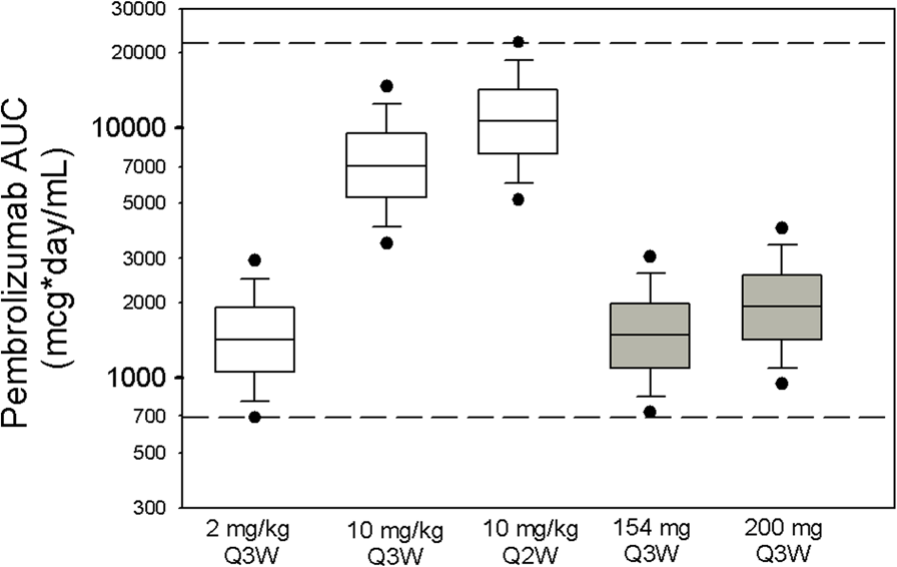
Simulated distribution of steady-state AUC exposures (2800 replicate simulations) for the weight-based regimens of 2 mg/kg Q3W, 10 mg/kg Q3W, and 10 mg/kg Q2W compared with the simulated distribution of exposures for two potential fixed-dose regimens (log scale): *Box*: *straight middle line* = median; *edges* = 25th and 75th percentiles; *whiskers* = 10th and 90th percentiles; *dots* = 5th and 95th percentiles. *Horizontal dashed lines* represent the range of exposures (5th percentile of 2 mg/kg Q3W and 95th percentile of 10 mg/kg Q2W) from dose regimens demonstrated to have comparable efficacy and tolerability in melanoma and NSCLC trials

The assessment of the potential to apply a fixed-dose regimen for pembrolizumab used a previously established population pharmacokinetics (popPK) model [4] and therapeutic window information derived from dose–response and exposure–response results in patients with advanced melanoma or non–small cell lung cancer (NSCLC) enrolled in KEYNOTE-001 [5], KEYNOTE-002 [6], and KEYNOTE-006 [7]. The mechanism of action of pembrolizumab, binding to PD-1 receptors on T cells, does not depend on direct engagement of the molecule with tumor cells. For this reason, substantial differences in exposure–response and dose–response are not expected across different tumor types. Indeed, it has been found that the pharmacokinetics (PK) of pembrolizumab are similar across oncology indications [4]. On this basis, selection of a fixed-dose regimen focused on establishing a dose that would provide comparable (central tendency and distribution) exposures as the 2 mg/kg Q3W regimen approved in the United States for melanoma and NSCLC. The fixed dose selected also aimed to maintain exposures within the existing clinical experience range that has been established for melanoma and NSCLC and which has been associated with a lack of clinically important differences in efficacy or safety (Fig. 1).

## Methods

### Clinical studies

Data to inform the fixed-dose evaluations were based on cross-study pooling from a number of ongoing and completed pembrolizumab clinical trials (Table 1). Further description of the trials are in Additional file 1: Table S1 including protocol numbers, title of the protocols and ClinicalTrials.gov identifier, with references to the published primary clinical results paper provided where available. These studies were conducted in accordance with the protocol, good clinical practice standards and the Declaration of Helsinki. The protocols and subsequent amendments were approved by the appropriate institutional review board (IRB) or ethics committee at each participating institution. All patients provided voluntary written informed consent.

**Table 1:** Number of patients with observed PK concentration data used in the analysis by study, cancer type and dosing regimen

Study	Cancer Type	2 mg/kg Q3W	10 mg/kg Q3W	10 mg/kg Q2W	200 mg Q3W
KEYNOTE-001	Melanoma	165	309	176	
KEYNOTE-001	NSCLC	61	288	204	
KEYNOTE-002	Melanoma	207	212		
KEYNOTE-006	Melanoma		270	272	
KEYNOTE-010	NSCLC	327	326		
KEYNOTE-024	NSCLC				152
KEYNOTE-045	Urothelial Cancer				262
KEYNOTE-052	Urothelial Cancer				311
KEYNOTE-055	HNSCC				47
KEYNOTE-164	MSI-H				58

There are patients with missing indication for *N* = 24 from KEYNOTE-001, 002 and 006

*NSCLC* Non–Small Cell Lung Cancer, *HNSCC* Head and Neck Squamous Cell Carcinoma, *MSI-H* Microsatellite Instability-High Carcinoma

The patients were treated with pembrolizumab in a dose range of 1–10 mg/kg administered as intravenous infusion, with the vast majority of the data collected under 4 dosing regimens (2 mg/kg Q3W, 10 mg/kg Q3W, 10 mg/kg Q2W, and 200 mg Q3W) as detailed in Table 1. Data used in this analysis included baseline patient characteristics (demographic factors, measure of renal and hepatic function, and measures of disease severity) and serum concentrations collected from these studies. Most pembrolizumab trials did not collect intensive (serial time-course) PK samples, which would allow for model-independent determination of PK parameter values such as area-under-the-curve (AUC) on a given study day. Rather sparse PK samples (1 or 2 samples per designated clinic visit) have been collected to minimize the burden on patients. The available concentration data were obtained either at peak (nominally within 30 min after end of infusion) or trough (nominally within 24 h before the next dose) samples and were obtained during pre-specified dosing cycles throughout the pembrolizumab treatment. Actual time of dosing and PK sampling were collected and used in the analyses. PK samples for pembrolizumab serum concentration determination were assayed using previously reported methods [4].

### Pharmacokinetics analysis

PopPK analysis was used to estimate PK parameters and exposures from observed sparse concentration data and to simulate PK under potential fixed dosing regimens, which informed the fixed dose selected for investigation in the trials. PopPK is a model-based approach to describe the time course of drug exposure across individuals in a population by estimation of both population-level typical PK values (eg, clearance, volume of distribution) and explicit terms to describe variability, including inter-subject variability, underlying the distribution of responses. It is the preferred method for interpreting sparse PK concentration data [8, 9].

A two-compartment pembrolizumab popPK model was used that was previously established based on data from KEYNOTE-001, -002 and -006 and is described in detail in [4]. The relationships between PK parameters (clearance [CL] and volume of distribution [Vc]) and body weight were estimated by the incorporation of an allometric exponential relationship with body-weight in the terms for these parameters:

1 $$ C L= C{L}_{TV}\cdot {\left(\frac{WT}{Median(WT)}\right)}^{\alpha - CL}\cdot {e}^{\eta_1} $$

2 $$ V c= V{c}_{TV}\cdot {\left(\frac{WT}{Median(WT)}\right)}^{\alpha - Vc}\cdot {e}^{\eta_2} $$

where X_TV_ is the typical value of the pharmacokinetic parameter X, and α-X is the allometric exponent describing the association with WT (individual body weight) normalized by MedianWT. The terms e^η^ described further inter-individual variation in these PK parameters beyond that accounted for by WT. Two additional parameters (Q and V_P_) described the distribution behavior of pembrolizumab and were also adjusted for WT, using the same values for the exponents as for CL and Vc, respectively. Covariate terms describing other factor associations were retained in the popPK model as identified in [4], and included sex, baseline estimated glomerular filtration rate (eGFR), baseline albumin, prior treatment with ipilimumab, cancer type, baseline Eastern Cooperative Oncology Group (ECOG) performance status, and baseline tumor burden (sum of longest dimensions of target lesions).

PK Estimation: The popPK model was re-estimated by fitting the previous dataset with the new KEYNOTE-10, KEYNOTE-055, KEYNOTE-024, KEYNOTE-164, KEYNOTE-045 and KEYNOTE-052 concentration data added to obtain individual post-hoc PK parameter estimates from which individual PK values were derived for AUC at steady state, over 6 weeks (AUC_ss_, _0-6weeks_), steady-state peak serum concentration (C_max, ss_) and steady-state trough serum concentration (C_trough, ss_). AUC_ss_, _0-6weeks_ was calculated as:

3 $$ A U C=\frac{Actual\; dose\;{(mg)}^{*}}{Clearance\;\left(\frac{L}{day}\right)}\times \frac{6(weeks)}{dosing\; interval(weeks)} $$

C_max, ss_ and C_trough, ss_ were determined from the concentration-time profile using each individual’s post-hoc estimated pharmacokinetic parameters. Summary statistics (mean, %CV, median, 10–90 percentiles) were determined by using R version 3.2.5 (Free Software Foundation, Boston, MA).

PK Simulation: To predict pembrolizumab PK under untested dosing regimens, virtual oncology patients were created by randomly drawing covariate values (body weight, albumin, bilirubin, baseline tumor burden, estimated glomerular filtration rate, sex, tumor type, baseline ECOG performance status, ipilimumab history) with replacement from the pooled baseline covariate data from available pembrolizumab studies (*n* = 3038 from KEYNOTE-001, -002, -006, -010, -011, -012, -025, -041 and -055) (Additional file 1: Table S1) to enable the simulation of exposures using the popPK model (2800 virtual patients simulated per each dosing group). Intersubject variability terms in the model were sampled from the established distributions, which together with fixed parameters (typical values and covariate relationships) determined parameter values (eg, CL, Vc) for each virtual patient, which were in turn used to determine PK values (AUC_ss_, _0-6weeks_, C_max, ss_, C_trough, ss_) as described above. Graphical plots were generated using R version 3.2.5 (Free Software Foundation, Boston, MA) and SigmaPlot 11.0 (Systat Software Inc., San Jose, CA).

### Drug product wastage calculation

To quantify the impact of a fixed-dosing regimen on drug product usage, the amount of remaining pembrolizumab product per single drug administration at 2 mg/kg Q3W using currently available 50- or 100-mg vials was calculated. First, the weight distribution in a typical oncology population was generated for 1000 subjects at random from the observed weight distribution in the popPK dataset (3.7% of ≤50 kg, 31.9% of >50–≤ 70 kg, 39.4% of >70–≤ 90 kg, 22.3% of >90–≤ 120 kg and 2.7% of >120 kg: Additional file 2: Table S2). The total amount of dose in mg for each subject was derived as the product of 2 mg/kg and body weight. Based on the total dose amount, the number of vials required was determined based on 50-mg or 100-mg vials available. The amount of remaining product for each subject per 2 mg/kg administration was calculated by the difference in the total amount available in the required vials and the total amount needed to dose at 2 mg/kg. Individual subject results were binned into 5 groups (0–10 mg, 10–20 mg, 20–30 mg, 30–40 mg, 40–50 mg for 50-mg vial, and 0–20 mg, 20–40 mg, 40–60 mg, 60–80 mg, 80–100 mg for 100-mg vial). Additionally, the overall amount of remaining drug product resulting from a full treatment course in these 1000 subjects was estimated by summing the product of the individual estimates for remaining product per dosing event and the typical number of doses received in a treatment course given the average pembrolizumab treatment duration of 6.2 months (~8 doses) (based on experience in early melanoma trials as described in label issued in September 2014).

## Results

### PopPK model and effect of body weight

Body weight is a known factor in drug exposure of therapeutic antibodies and therefore the weight-dependency of the PK properties was robustly evaluated in the popPK modeling efforts. The dataset used in a previously reported popPK analysis included 1622 patients (73.9%) with advanced melanoma, 551 patients (25.1%) with advanced NSCLC, and 22 patients (1.0%) with other advanced malignancies [4] from KEYNOTE-001, -002 and -006. The analysis population encompassed a wide distribution of body weight, with a median of 77.2 kg and a range of 35.7–209.5 kg. Estimates (90% confidence intervals [CI]) of the relationship between clearance and body weight based on the popPK model revealed an allometric exponent (α) of 0.578 (95% CI, 0.481–0.666) for the clearance parameters and 0.492 (95% CI, 0.432–0.553) for the volumes of distribution. Theoretically, fixed dosing would work the best when CL is not affected by body weight (α = 0) and body-weight–based dosing would work the best when α equals to 1 [1–3]. Given that α estimates were close to 0.5 for both clearance and volume of distribution, no advantage of weight-based dosing over fixed dosing is expected, and both weight-based and fixed dosing should provide adequate and similar control of PK variability.

### Simulation results informing selection of 200 mg Q3W as fixed dose for investigation in trials

The expected distributions of pembrolizumab exposures from potential fixed doses administered Q3W were simulated using the popPK model and compared with the distributions expected from the weight-based dosing regimens (2 mg/kg Q3W, 10 mg/kg Q3W, and 10 mg/kg Q2W) studied in the melanoma and NSCLC trials that supported the current US registrations. The distribution of exposures from these weight-based regimens represents the range of clinical experience to date, where the safety profile, overall response rate and survival outcomes have been found to be similar across the tested dosing regimens in the melanoma and NSCLC trials [5–7, 10, 11], and a flat exposure-response relationship was identified across these three regimens in evaluations of tumor size response [12, 13] and immune-related adverse events [6, 7]. Based on this flat dose- and exposure-response relationship, 2 mg/kg Q3W regimen was initially approved in the United States for melanoma and NSCLC as a dosing regimen which achieves clinically meaningful efficacy with limited additional clinical benefit at higher dose levels. Figure 1 displays the distribution of steady-state AUC exposures predicted by the popPK model. The AUC values are displayed on a log scale, allowing for ready comparison of the relative PK variability with weight-based versus fixed dosing. As expected based on the allometric exponent values discussed above, the PK variability from weight-based and fixed-dose regimens is nearly identical. A fixed dosage of 154 mg Q3W was identified as providing almost identical steady-state AUC exposure as a weight-based dosage of 2 mg/kg Q3W. The distribution of exposures from 200 mg Q3W substantially overlaps that obtained with the 2 mg/kg Q3W dose and is well within the exposure range associated with maximal clinical response and acceptable tolerability in melanoma and NSCLC.

**Fig. 2:**
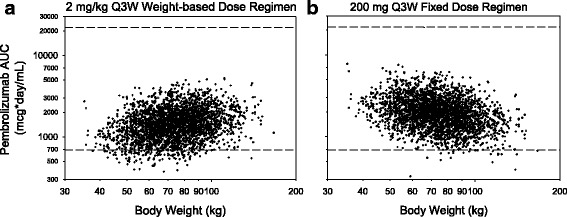
Predicted variation in pembrolizumab AUC exposure by body weight for weight-based (**a**) and fixed-dose (**b**) regimens (2800 replicate simulations): *Horizontal dashed lines* represent the range of exposures (5th percentile of 2 mg/kg Q3W and 95th percentile of 10 mg/kg Q2W) from dose regimens demonstrated to have comparable efficacy and tolerability in melanoma and NSCLC trials

The predicted variation in pembrolizumab AUC exposure with patient body weight for the 2 mg/kg Q3W and 200 mg Q3W regimens is shown in Fig. 2. For the weight-based regimen, lower-weight patients tend to have lower exposures relative to higher-weight patients, while the opposite trend is seen with fixed dosing. For both regimens, the range of individual exposures for the low-weight patients considerably overlaps that for the high-weight patients, consistent with PK variability being only partially explained by weight. The overall extent of PK variability appears similar for both regimens. The 200 mg Q3W regimen was selected for investigation in the clinical trials based on the similarity of the exposures to 2 mg/kg with a slight upward shift to ensure individual patient exposures, especially in patients with a higher weight, fall within the range of prior clinical experience. Of note, with the 200 mg Q3W regimen, very few simulated individual patients fell below the fifth percentile of exposures from the approved 2 mg/kg Q3W regimen. Both regimens were predicted to yield a range of exposures that falls well below the highest exposure for which acceptable tolerability has been demonstrated.

### Observed 200 mg Q3W fixed-dose exposures

**Fig. 3:**
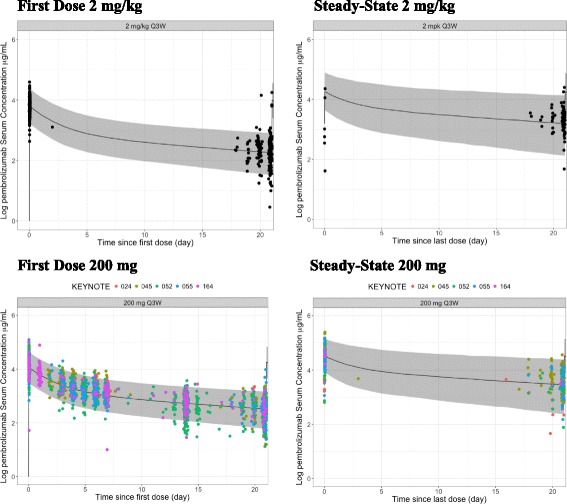
Consistency of observed concentrations in patients with predictions based on population PK model: Pembrolizumab concentration-time profiles during the first dose (*left panels*) and at steady state (*right panels*) of repeated dosing at 2 mg/kg Q3W (*top panels*) and 200 mg Q3W (*bottom panels*). *Solid markers* represent observed pembrolizumab serum concentrations. *Solid line* represents median predicted concentration time profile, based on the population PK model. *Shaded areas* represent 90% prediction interval for the prediction

**Fig. 4:**
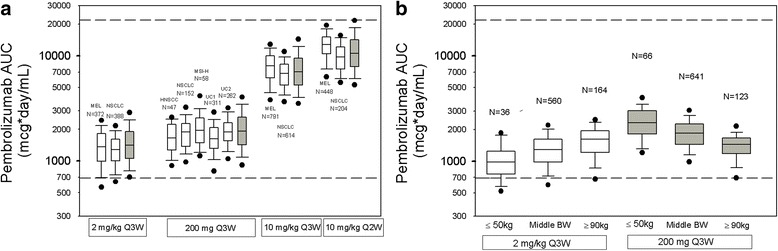
Distribution of observed pembrolizumab AUC_ss_, _0-6weeks_: Panel **a** – Consistency with model predictions (Simulated values shown in *gray* and observed values in *white*). Panel **b** – Variation in exposures with body weight under weight-based versus fixed dosing. *Box*: *straight middle line* = median; *edges* = 25th and 75th percentiles; *whiskers* = 10th and 90th percentiles; *dots* = 5th and 95th percentiles. *Horizontal dashed lines* (------) represent the range of exposures (5th percentile of 2 mg/kg Q3W and 95th percentile of 10 mg/kg Q2W) from dose regimens demonstrated to have comparable efficacy and tolerability in melanoma and previously treated NSCLC trials. Observed data are based on Table 1. In Panel B, distribution of observed AUC_ss_, _0-6weeks_ for light (≤50 kg), middle (between 50 and 90 kg) and heavy (≥90 kg) body-weight patients

**Fig. 5:**
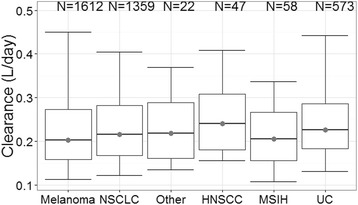
Consistency of pembrolizumab clearance in patients with differing cancer: melanoma from KEYNOTE-001, -002 and -006. NSCLC from KEYNOTE-001, -010 and -024. Other (other cancers) from KEYNOTE-001 in initial cohort. HNSCC (head and neck trial) from KEYNOTE-055. MSIH (MSI-H in CRC) from KEYNOTE-164. UC (urothelial cancer trial) from KEYNOTE-045 and -052

Observed PK data for 200 mg Q3W fixed dosing from patients with head and neck cancer, NSCLC, MSI-H in CRC and urothelial cancer treated with pembrolizumab in KEYNOTE-055, -024, -164, -52 and -045, respectively, confirm the exposure predicted for this regimen based on the popPK model. The observed concentration data from 200 mg Q3W are consistent with the model-predicted time course of concentration over the dosing interval both early in therapy and after PK steady-state is achieved (Fig. 3). Figure 3 also illustrates that the shape of the PK concentration-time profile with the fixed-dose regimen is similar to that obtained with the 2 mg/kg regimen in the earlier trials. The AUC exposures obtained in the 200 mg Q3W trials also indicate a good match of observed and predicted PK, with the distribution of observed exposures falling within the range of previous clinical experience derived from the weight-based regimens (Fig. 4a). In this analysis, PK data were obtained in patients with several cancer types not previously described. Clearance values across all cancer types were not meaningfully different (Fig. 5), supporting the consistency of pembrolizumab PK across cancer types.

Summary statistics for the observed pembrolizumab exposures across the 4 dosing regimens (Table 2) indicate that the central tendency (mean, median) at 200 mg Q3W is modestly increased (~35%) relative to 2 mg/kg Q3W for all PK measures (AUC_ss_, _0-6weeks_, C_max, ss_ and C_trough, ss_), while these values are ~25% of those obtained at 10 mg/kg Q3W. Intersubject variation (% CV) is similar for all regimens and the 10–90% percentiles are largely overlapping for 2 mg/kg and 200 mg Q3W. The distribution of observed exposures with the 2 mg/kg and 200 mg Q3W regimens were compared among three weight-based subpopulations: light (body weight ≤50 kg), middle (body weight between 50 and 90 kg) and heavy (body weight ≥90 kg) to investigate the influence of extreme body weights on exposures (Fig. 4b). The distribution of body weights in the patients studied under these two regimens was similar (Additional file 3: Figure S1). The influence of body weight trended as predicted in the simulations. Although heavier patients had lower exposures with the 200 mg fixed dose, the distribution of exposures obtained in these patients was contained within the range of exposures from the prior clinical experience.

**Table 2:** Pharmacokinetics of pembrolizumab at steady state of regimens of 2 mg/kg Q3W, 200 mg Q3W, 10 mg/kg Q3W and 10 mg/kg Q2W. Based on pooled cross-study data [n, Mean (%CV), Median (10-90 percentile)]

PK Value (unit)	Dose Regimen	N	Mean (%CV)	Median	10–90 Percentile
C_max_ (mcg/mL)	2 mg/kg Q3W	755	68.0 (24%)	66.3	48.3–88.2
	200 mg Q3W	830	93.4 (26%)	89.1	66.4–124.3
	10 mg/kg Q3W	1403	360.3 (23%)	357.6	257.7–466.8
	10 mg/kg Q2W	652	459.3 (25%)	457.7	315.9–599.9
C_trough_ (mcg/mL)	2 mg/kg Q3W	755	22.2 (48%)	21.1	9.18–35.7
	200 mg Q3W	830	29.7 (47%)	27.6	14.9–46.2
	10 mg/kg Q3W	1403	126.4 (44%)	120.4	59.8–200.2
	10 mg/kg Q2W	652	220.9 (39%)	217.8	111.8–325.3
AUC_ss, 0-6-weeks_ (mcg*day/mL)	2 mg/kg Q3W	760	1376.5 (38%)	1316.5	724.9–2038.5
	200 mg Q3W	830	1871.1 (37%)	1787.0	1120.6–2730.9
	10 mg/kg Q3W	1405	7625.4 (35%)	7436.0	4354.0–11172.8
	10 mg/kg Q2W	652	12002.7 (34%)	11993.5	6834.7–16895.5

### Discarded amount with weight-based dosing

The amount of remaining pembrolizumab product from administration of a 2 mg/kg weight-based dose using 50- or 100-mg vials was estimated based on the distribution of body weight in the analysis dataset (Additional file 4: Table S3). On average, 27 and 56 mg per patient per administration would remain when using 50- and 100-mg vials, respectively. Given that the average pembrolizumab treatment duration was 6.2 months (approximately eight doses) in patients with melanoma treated in KEYNOTE-001, approximately 220 g or 450 g pembrolizumab would be remaining for every 1000 patients treated using the weight-based dosing with the 50- or 100-mg vials, respectively. By contrast, no remaining product is expected at 200-mg fixed dosing using 50- or 100-mg vials.

## Discussion

The evaluations and illustrations provided in this paper provide an example that for mAbs, there is no set answer as to whether weight-based or fixed-dose strategies are better. Therefore, the appropriate dosing strategy should be evaluated based on the PK properties of the given mAb. It was demonstrated in this paper that both weight-based and fixed dosing are appropriate for pembrolizumab, with neither regimen providing a PK advantage over the other. Although there is no PK advantage for either regimen, fixed dosing would eliminate the waste generated by weight-based dosing, improve compliance and might also reduce the risk of dosing errors by reducing dosing complexity.

Pembrolizumab is currently available in 50- or 100-mg vials. When using a weight-based dosing regimen, the contents of the final vial are generally incompletely administered, and the remaining drug product is discarded as per labeling instructions. In practice it might potentially be used for another patient, raising quality concerns and, consequently, potential safety concerns as it represents a source of infection when it is used inappropriately outside of the clinical trial setting. Centers for Disease Control and Prevention (CDC) reported several instances including mishandling of injectable medications such as reuse of single-dose vials for more than one patient. From 2010–2014, CDC is aware of at least 26 outbreaks due to unsafe injection practices. These outbreaks resulted in more than 95,000 patients being referred for testing after potential exposure to infectious diseases. 73% (*n* = 19) of these outbreaks involved use of single-dose/single-use medications for more than one patient [14, 15].

The results presented in this paper demonstrate that fixed dosing of pembrolizumab 200 mg Q3W maintains exposures comparable with or slightly increased relative to those from 2 mg/kg Q3W (the initially approved dose for pembrolizumab). All patients, including high-weight patients, achieve exposures in the range which has been demonstrated in clinical dose-ranging trials to provide near maximal efficacy. Exposures achieved by 200 mg Q3W also fall well below the high dose clinical experience at 10 mg/kg for which acceptable tolerability has been demonstrated. Exposures which match or exceed those at 2 mg/kg also ensure that maximal target engagement is achieved as informed by early PK/PD work with a clinical biomarker (IL-2 release) in KEYNOTE-001 which demonstrated saturation of response at 1 mg/kg [16, 17]. The estimated median clearance of pembrolizumab in the patients receiving 200 mg Q3W was 0.22 L/day, which is similar to the 0.23 L/day obtained in patients receiving 2 mg/kg Q3W as well as the clearance of endogenous IgG (0.21 L/day) and consistent with linear clearance characteristics of typical mAbs (0.2–0.5 L/day) [18, 19]. The consistency of PK across cancer types supports the use 200 mg in Q3W in various cancer types.

Recently, 200 mg Q3W was approved for use in patients with NSCLC [20] and HNSCC [21–23] in the United States, and clinical results show similar efficacy and safety in these indications among doses (2 mg/kg Q3W, 200 mg Q3W, 10 mg/kg Q3W/Q2W) in the trials supporting these indications.

## Conclusions

In conclusion, the 200 mg Q3W fixed dosage can be considered an appropriate fixed-dose regimen for pembrolizumab based on the achievement of exposures well within the prior clinical experience demonstrated to be associated with near maximal efficacy and acceptable tolerability. The 200 mg Q3W dose of pembrolizumab, which continues to be investigated in trials for various oncology indications, may also be an appropriate alternative for patients currently being treated with the approved 2 mg/kg Q3W dose.

## Additional files

Additional file 1:Table S1. Description of clinical trials used in analyses [24]. (DOCX 13 kb)

Additional file 2: Table S2. Body weight distribution in the population PK analysis dataset (*N* = 2195; KEYNOTE-001 + KEYNOTE-002 + KEYNOTE-006). (DOCX 12 kb)

Additional file 3: Figure S1. Observed body weight distribution for 2 mg/kg Q3W and 200 mg Q3W. Observed weight distribution of total *N* = 1591 (*N* = 760 who received 2 mg/kg Q3W and *N* = 830 who received 200 mg Q3W). KEYNOTE-001, -002, -010 at 2 mg/kg Q3W, -024, -052, -055, -045 and -164 at 200 mg Q3W (KEYNOTE-006 contains only 10 mg/kg). Median weights: 74.0 kg for 2 mg/kg Q3W (solid red line) and 71.8 kg for 200 mg Q3W (solid blue line). Black dot lines: 50 kg and 90 kg. (PNG 6 kb)

Additional file 4: Table S3. Distribution of patients as a function of amount (mg) remaining product per one administration at 2 mg/kg using 50-mg or 100-mg vial. The amount of remaining product was categorized into 5 groups (0–10 mg, 10–20 mg, 20–30 mg, 30–40 mg, 40–50 mg for 50-mg vial, and 0–20 mg, 20–40 mg, 40–60 mg, 60–80 mg, 80–100 mg for 100-mg vial) and the distribution of patients by these categories and the total amount of remaining drug product associated with weight-based dosing. Approximately 20% of the population would fall in each category. (DOCX 13 kb)

## Abbreviations

%CV: Coefficient of variation of between-subject variability of parameters
α: Allometric exponent
AUC: Area-under-the-curve
AUCss: _0–6-weeks_, Area under the concentration-time curve at steady state over a 6-week interval
CDC: Centers for Disease Control and Prevention
CI: Confidence interval
CL: Clearance
C_max, ss_: Steady-state maximum observed serum concentration
CRC: Colorectal cancer
C_trough, ss_: Steady-state concentration at the end of the dosing interval
EGFR: Epidermal growth factor receptor
eGFR: Estimated glomerular filtration rate
HNSCC: Head and neck squamous cell carcinoma
IgG: Immunoglobulin G
IL-2: Interleukin-2
IRB: Institutional review board
mAbs: Monoclonal antibodies
MEL: Melanoma
MSI-H: Microsatellite Instability High
N: Number
NSCLC: Non–small cell lung cancer
PD-1: Programmed death 1
PD-L1: Programmed death ligand 1
PK: Pharmacokinetics
popPK: Population pharmacokinetic
Q: Intercompartmental clearance
Q2W: Every 2 weeks
Q3W: Every 3 weeks
UC: Urothelial cancer
Vc: Central volume of distribution
Vp: Peripheral volume of distribution
