# Supplementary material for: Evaluation of dosing strategy for pembrolizumab for oncology indications
Source: J Immunother Cancer. 2017 May 16;5:43. doi: 10.1186/s40425-017-0242-5 (PMC5433037; doi:10.1186/s40425-017-0242-5)
Supplement: Supplementary file 1 — Description of clinical trials used in analyses [24]. (DOCX 13 kb) [file 40425_2017_242_MOESM1_ESM.docx]

Table S1 Description of clinical trials used in analyses

| Protocol # | Title^Ref^ | ClinicalTrials.gov identifier |  |
| --- | --- | --- | --- |
| KEYNOTE-001 (KN001) | Phase I Study of Single Agent Pembrolizumab (MK-3475) in Patients with Progressive Locally Advanced or Metastatic Carcinoma, Melanoma, and Non-Small Cell Lung Carcinoma^5^ | NCT01295827 |  |
| KEYNOTE--002 (KN002) | Randomized, Phase II Study of MK-3475 versus Chemotherapy in Patients with Advanced Melanoma^6^ | NCT01704287 |  |
| KEYNOTE--006 (KN006) | A Multicenter, Randomized, Controlled, Three-Arm, Phase III Study to Evaluate the Safety and Efficacy of Two Dosing Schedules of MK-3475 Compared to Ipilimumab in Patients with Advanced Melanoma^7^ | NCT01866319 |  |
| KEYNOTE--010 (KN010) | A Phase II/III Randomized Trial of Two Doses of MK-3475 (SCH900475) versus Docetaxel in Previously Treated Subjects with Non-Small Cell Lung Cancer^24^ | NCT01905657 |  |
| KEYNOTE--011 (KN011) | A Phase I Study of MK-3475 Alone in Subjects with Advanced Solid Tumors and in Combination with Cisplatin/Pemetrexed or Carboplatin/Pemetrexed in Subjects with Advanced Non-Small Cell Lung Cancer (in Japanese only) | NCT01840579 |  |
| KEYNOTE--012 (KN012) | Study of Pembrolizumab (MK-3475) in Participants With Advanced Solid Tumors^21, 22^ | NCT01848834 |  |
| KEYNOTE--025 (KN025) | An open-label, non-randomized, multi-center Phase Ib Study of MK-3475 in Subjects with  PD-L1 positive Advanced Non-Small Cell Lung Cancer (in Japanese only) | NCT02007070 |  |
| KEYNOTE--041 (KN041) | Phase Ib Study of MK-3475 in Subjects with Advanced Melanoma (in Japanese only) | NCT02180061 |  |
| KEYNOTE--055 (KN055) | A Phase II Clinical Trial of Single Agent Pembrolizumab (MK-3475) in Subjects with Recurrent or Metastatic Head and Neck Squamous Cell Carcinoma (HNSCC) Who Have Failed Platinum and Cetuximab^23^ | NCT02255097 |  |
| KEYNOTE--024 (KN024) | A Randomized Open-Label Phase III Trial of MK-3475 versus Platinum based Chemotherapy in 1L Subjects with PD-L1 Strong Metastatic Non-Small Cell Lung Cancer^20^ | NCT02142738 |  |
| KEYNOTE--164 (KN164) | A Phase II Study of Pembrolizumab (MK-3475) as Monotherapy in Subjects with Previously Treated Locally Advanced Unresectable or Metastatic (Stage IV) Mismatched Repair Deficient or Microsatellite Instability-High Colorectal Carcinoma | NCT02460198 |  |
| KEYNOTE—052 (KN052) | A Phase II Clinical Trial of Pembrolizumab (MK-3475) in Subjects with Advanced/Unresectable or Metastatic Urothelial Cancer | NCT02335424 |  |
| KEYNOTE--045 (KN045) | A Phase III Randomized Clinical Trial of Pembrolizumab (MK-3475) versus Paclitaxel, Docetaxel or Vinflunine in Subjects with Recurrent or Progressive Metastatic Urothelial Cancer | NCT02256436 |  |
