# Supplementary material for: Evaluation of dosing strategy for pembrolizumab for oncology indications
Source: J Immunother Cancer. 2017 May 16;5:43. doi: 10.1186/s40425-017-0242-5 (PMC5433037; doi:10.1186/s40425-017-0242-5)
Supplement: Supplementary file 2 — Body weight distribution in the population PK analysis dataset (N = 2195; KEYNOTE-001 + KEYNOTE-002 + KEYNOTE-006). (DOCX 12 kb) [file 40425_2017_242_MOESM2_ESM.docx]

Table S2 Body Weight Distribution in the Population PK Analysis Dataset (*N* = 2195; KEYNOTE-001 + KEYNOTE-002 + KEYNOTE-006)

| Weight Categories | Number of Patients | Percentage |
| --- | --- | --- |
| ≤50 kg | 82 | 3.74 % |
| >50 kg to ≤70 kg | 699 | 31.85 % |
| >70 kg to ≤90 kg | 864 | 39.36 % |
| >90 kg to ≤120 kg | 490 | 22.32 % |
| >120 kg | 60 | 2.73 % |
| Total | N=2195 | 100 % |

*N* = 2195 subjects from KEYNOTE-001, KEYNOTE-002, and KEYNOTE-006
