# Supplementary figures and images for: Evaluation of dosing strategy for pembrolizumab for oncology indications
Source: J Immunother Cancer. 2017 May 16;5:43. doi: 10.1186/s40425-017-0242-5 (PMC5433037; doi:10.1186/s40425-017-0242-5)

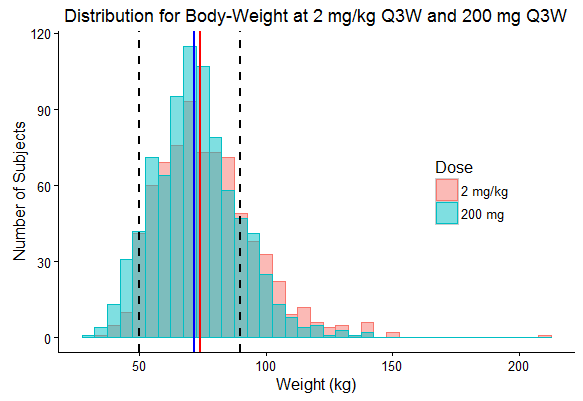

Supplement: Supplementary file 3 — Observed body weight distribution for 2 mg/kg Q3W and 200 mg Q3W. Observed weight distribution of total N = 1591 (N = 760 who received 2 mg/kg Q3W and N = 830 who received 200 mg Q3W). KEYNOTE-001, -002, -010 at 2 mg/kg Q3W, -024, -052, -055, -045 and -164 at 200 mg Q3W (KEYNOTE-006 contains only 10 mg/kg). Median weights: 74.0 kg for 2 mg/kg Q3W (solid red line) and 71.8 kg for 200 mg Q3W (solid blue line). Black dot lines: 50 kg and 90 kg. (PNG 6 kb) [file 40425_2017_242_MOESM3_ESM.png]
