# Supplementary material for: Evaluation of dosing strategy for pembrolizumab for oncology indications
Source: J Immunother Cancer. 2017 May 16;5:43. doi: 10.1186/s40425-017-0242-5 (PMC5433037; doi:10.1186/s40425-017-0242-5)
Supplement: Supplementary file 4 — Distribution of patients as a function of amount (mg) remaining product per one administration at 2 mg/kg using 50-mg or 100-mg vial. The amount of remaining product was categorized into 5 groups (0–10 mg, 10–20 mg, 20–30 mg, 30–40 mg, 40–50 mg for 50-mg vial, and 0–20 mg, 20–40 mg, 40–60 mg, 60–80 mg, 80–100 mg for 100-mg vial) and the distribution of patients by these categories and the total amount of remaining drug product associated with weight-based dosing. Approximately 20% of the population would fall in each category. (DOCX 13 kb) [file 40425_2017_242_MOESM4_ESM.docx]

**Table S3** **Distribution of patients as a function of amount (mg) remaining product per one administration at 2 mg/kg using 50-mg or 100-mg vial**

**50-mg Vial and 100 mg Vial**

| **50 mg Vials** | | **100 mg Vials** | |
| --- | --- | --- | --- |
| **Number of Patients (%)** | **Total amount remaining produce** | **Number of Patients (%)** | **Total amount remaining product** |
| **156 (15.6%)** | 776.75 mg | **94 (9.4%)** | 881.75 mg |
| **159 (15.9%)** | 2340.08 mg | **214 (21.4%)** | 6286.35 mg |
| **235 (23.5%)** | 5868.28 mg | **214 (21.4%)** | 10682.94 mg |
| **228 (22.8%)** | 7939.00 mg | **233 (23.3%)** | 16359.21 mg |
| **222 (22.2%)** | 10028.55 mg | **245 (24.5%)** | 21992.42 mg |
| **N=1000** | **26952.66 mg** | **N=1000** | **56202.67 mg** |

Amount of remaining per 1 administration for 1000 subjects at 2 mg/kg Q3W

The amount of remaining product was categorized into 5 groups (0–10 mg, 10–20 mg, 20–30 mg, 30–40 mg, 40–50 mg for 50-mg vial, and 0–20 mg, 20–40 mg, 40–60 mg, 60–80 mg, 80–100 mg for 100-mg vial) and the distribution of patients by these categories and the total amount of remaining drug product associated with weight-based dosing. Approximately 20 % of the population would fall in each category.
